# Learning from inborn errors of immunity and secondary immune deficiencies about vaccine immunogenicity, efficacy, and safety

**DOI:** 10.1093/cei/uxaf059

**Published:** 2025-09-02

**Authors:** Nicholas E Peters, Adrian M Shields, Sophie Hambleton, Alex G Richter

**Affiliations:** Clinical Immunology Service, Institute of Immunology and Immunotherapy, University of Birmingham, Birmingham, UK; Clinical Immunology Service, Institute of Immunology and Immunotherapy, University of Birmingham, Birmingham, UK; Translational and Clinical Research Institute, Faculty of Medical Sciences, Newcastle University, Newcastle upon Tyne, UK; Clinical Immunology Service, Institute of Immunology and Immunotherapy, University of Birmingham, Birmingham, UK

**Keywords:** vaccine, immunodeficiency diseases, infection

## Abstract

Since its discovery in the late 18th century, the role of vaccination in preventing death and disease has expanded across many infectious diseases and cancer. Key to our understanding of vaccine immunogenicity and efficacy is knowledge of the immune system itself. Inborn errors of immunity (IEI) represent a heterogeneous group of disorders characterized by impaired function of the immune system. Patients with IEI can have variable responses to vaccinations, depending on the nature and extent of the defect. Studies performed during the recent COVID-19 pandemic have brought unique insight into vaccine immunogenicity in individuals with IEI, knowledge that can be extended to the growing number of patients with secondary immunodeficiency arising from malignancy, organ transplantation, autoimmune conditions, and their treatments. In this review, we describe vaccine immunogenicity in IEI alongside their equivalent secondary immunodeficiencies and discuss what lessons can be learned about immunization strategies more broadly.

## Teaser

In this review, we describe vaccine immunogenicity in IEI alongside their equivalent secondary immunodeficiencies and discuss what lessons can be learned about immunisation strategies more broadly.

## Graphical Abstract

**Graphical Abstract:**
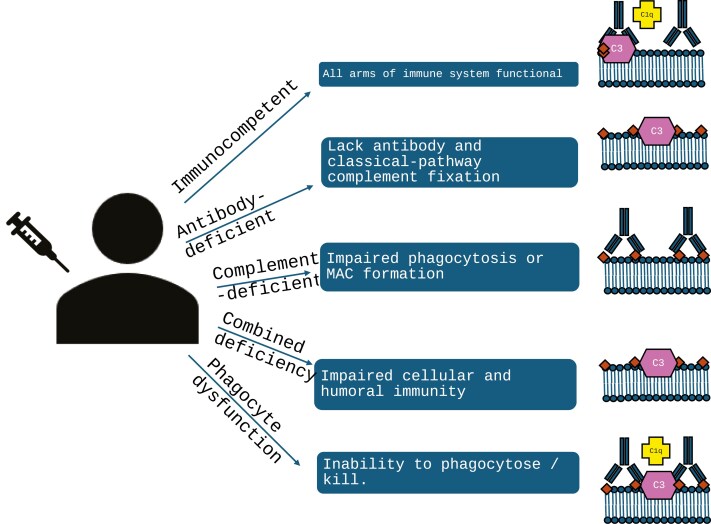

## Introduction

The World Health Organization estimates that vaccination has averted 154 million deaths over the last 50 years since the launch of the Expanded Programme on Immunization with stunning successes including the eradication of smallpox [1]. Vaccination remains one of the most impactful public health interventions, supported by diverse platforms ranging from live attenuated organisms to the more recent mRNA vaccines. Regardless of the vaccine type, a functioning immune system is crucial for the recipient to mount a response and develop long-term immunological memory in the absence of primary infection [2].

Inborn errors of immunity (IEI) comprise a heterogeneous group of over 550 genetic disorders that impair immune function, as documented by the 2024 International Union of Immunological Societies phenotypic classification [3]. These defects not only predispose individuals to specific infections but also impact their ability to respond effectively to vaccines. Depending on the nature and severity of the immune defect, IEI can result in poor vaccine immunogenicity, reduced durability of immunity, or, in the case of live vaccines, an increased risk of vaccine-related complications [4], summarized in Table 1.

**Table 1:** summary of IEI with infection susceptibilities and predicted or observed effects on vaccine efficacy and safety

Inborn error of immunity category	Infection susceptibility	IEI-specific vaccine safety concerns	Vaccine efficacy and recommendations
**Phagocyte defects, e.g. CGD**	Bacterial, e.g. *S. aureus*, *Nocardia spp*, *Esherichia coli*, *Serratia marcescens*, *Salmonella spp* Fungal, e.g. *Aspergillus fumigatus*	Live attenuated bacterial vaccines, e.g. BCG, Salmonella can result in disseminated disease	Routine non live vaccinations recommended.
**Complement Deficiency, e.g. C1q deficiency, Terminal complement component deficiency (C5-C9)**	Terminal pathway components (membrane attack complex) C5–C9 Neisserial species	No specific safety concerns with current vaccines	Vaccines are able to elicit pathogen-specific adaptive immune responses; however, the effector function in the absence of complement is impaired. Lack of complement deposition may lead to impaired co-stimulatory signals to lymphocytes. Vaccine-strain breakthrough infections with meningococci have been reported in patients with terminal pathway deficiencies postvaccination, so prophylactic antibiotics are still recommended.
	Classical / alternative pathway defects—encapsulated bacteria, e.g. *S. pneumoniae* and Neisserial species		
	Alternative pathway components		
**Cell intrinsic signalling, e.g. IFN pathway deficiencies, TLR pathway deficiencies**	Interferon pathway signalling—multiple viral infections	Live viral vaccines may induce disease, e.g. MMR, Yellow fever virus vaccine	Adaptive immune response to microorganisms may compensate for innate immune defect, for at least some TLR defects, suggesting generation of high affinity antibodies possible and long-lived memory following vaccination. Routine nonlive vaccinations recommended.
	TLR pathway signalling defects, e.g. IRAK-4/MyD88—*Streptococcus pneumonia*, *S. aureus*, *P. aeruginosa*	Safety concerns with NF-κB defects for live vaccines.	
**Predominant antibody deficiency, e.g. CVID, XLA**	Encapsulated bacteria, e.g. *S. pneumoniae*, *H. influenzae*, *Moraxella catarrhalis* Viral infections, e.g. Norovirus, SARS-CoV-2,	Live viral vaccines—long term carriage of vaccine-strain poliovirus reported in antibody-deficient patients, vaccine strain rubella can cause granulomatous disease in antibody deficient patients	Some states of antibody deficiency may allow for partial compensatory protection and so routine vaccines recommended despite risk of reduced effectiveness. XLA—no antibody response but T cell responses noted CVID—Variable T cell responses. Antibody responses possible in some patients, e.g. if additional doses of vaccine given.
**Combined immunodeficiencies**	Multiple infection susceptibilities across bacteria, viruses, and fungi	All live vaccines	Reduced vaccine responsiveness due to T cell defect, therefore reduced response to T cell-dependent antigens. Nonlive vaccinations recommended but risk of reduced effectiveness
**Severe combined immunodeficiencies**	Multiple infection susceptibilities across bacteria, viruses, and fungi	All live vaccines	Vaccine efficacy not studied, very unlikely to be of any benefit. BCG vaccination for general population delayed due to risk in specific IEI.

Studying vaccine responses in rare IEI provides a lens for understanding the mechanisms underlying vaccine-induced immunity and subsequent protection from infectious disease. While the rarity of most IEI limits large-scale studies, considering vaccine responses across groups of IEI, categorized by their effects on components of innate immunity, B cells and antibodies, or T cells, provides meaningful insights. These lessons extend beyond IEI, offering valuable strategies for optimizing vaccination in patients with acquired or secondary immunodeficiencies, such as those undergoing treatment for malignancy, organ transplantation, or autoimmune conditions.

Key to understanding vaccine responses in a variety of immunodeficiencies is understanding how these responses are measured. A broad variety of laboratory techniques are employed to evaluate these responses but typically look at one aspect of the immune response. A summary is provided in Table 2. The measurement of immunogenicity indicates the success of a vaccine from the perspective of the particular component of the immune system being assessed; however, patients with an IEI affecting a downstream effector mechanism may not derive full efficacy, despite immunogenicity.

**Table 2:** overview of techniques used to assess immune responses to vaccines/pathogens

Assay	How the assay is performed	What is measured	Comments/Drawbacks in assessing vaccine responses in patients with IEI
Antibody quantification using ELISA	Incubation of serum with antigens bound to surface. Secondary reactions lead to a quantitative readout of optical density	Serum antibody titre	Routinely available in clinical laboratories, however does not provide information regarding functionality
Serum bactericidal assay [5]	Heat-inactivated patient serum is mixed with target bacteria and additional source of complement added (e.g. rabbit serum). Response measured by bacterial killing	Bacterial killing by complement. Functionality of antibody when mixed with exogenous serum.	A better indicator of complement fixation on live bacteria of a given antibody titre generated by ELISA. Not routinely clinically available. Addition of exogenous complement source might not reflect *in vivo* killing in complement-deficient patients or patients with phagocyte disorders.
Opsonophagocytic assay [6]	Serum is incubated bacteria (e.g. producing a reporter protein such as luciferase), exogenous complement (e.g. rabbit) and a phagocyte cell line. Surviving bacteria are quantified using plating, luminescence or colorimetry/turbidimetry.	Bacterial killing by complement/phagocytosis.	Not routinely clinically available. Addition of exogenous complement source and non-patient phagocytes might not reflect *in vivo* killing in complement-deficient patients or patients with phagocyte disorders. Better indicator of serum antibody to fix complement and induce phagocytosis.
Neutralization assay [7]	Serum is incubated with live virus and then added to susceptible cell lines. Readout is cytopathic effects on cell line	Ability of serum antibody to block infection of cells	Serum antibody has additional functions beyond neutralization
Pseudoneutralization assay [7]	Serum is incubated with pseudovirus bearing surface proteins of	Ability of serum antibody to block infection of cells	Safer laboratory technique that doesn’t require intact whole virus. May be used for emerging pathogens. Does not reflect all effects of serum antibody
B cell tetramer assay [8]	Antigens are conjugated to fluorescent labels and incubated with patient blood. Flow cytometry is used to identify B cells recognizing the given antigen	Antigen-specific B cells	Can be used to identify vaccine responsiveness in patients receiving immunoglobulin replacement therapy. Does not necessarily indicate production of antibody
Interferon gamma release assay [9]	Whole blood is stimulated with antigens, e.g. peptide libraries. Released interferon gamma from antigen-specific T cells is then measured.	Interferon gamma production	Clinically available for small number of pathogens (e.g. *Mycobacterium tuberculosis*). May be useful in determining cellular response to vaccines in antibody-deficiency patients. Less standardized than humoral assays without clear immune correlates of protection. Production of IFN-γ may not reflect utility e.g. in patients lacking IFN-γ receptor, or IL-12p40/STAT1 defects Immune correlates of protection are not adequately established for cellular assays at present

## Defects of innate immunity

The innate immune system is an evolutionarily ancient network of cells and proteins that provides rapid responses to tissue insult to preserve tissue homeostasis. Vaccines rely on the innate immune system to initiate acute inflammation thereby recruiting immune cells and shape subsequent adaptive immune responses [3, 10, 11]. Exploring how defects in components of the innate immune system (including complement proteins, intracellular signalling pathways, cytokines, and phagocytes) influence susceptibility to specific infections, and shape vaccination responses, provides valuable insights into vaccine efficacy and safety and may guide vaccine design, optimizing immunogenicity [12–14].

## Complement proteins

The complement system consists of plasma proteins that facilitate pathogen opsonization, enhance phagocytosis, and form membrane attack complexes (MAC) to lyse target cells [10, 15]. Genetic defects in complement activation pathways lead to varying infection susceptibilities. For example, individuals with autosomal recessive deficiencies in MAC components (C5-C9) are prone to infections by Neisserial species, especially *Neisseria meningitidis* [11], but do not appear to be susceptible to other bacterial infections. It is not clear from a microbiological perspective why deficiencies in MAC components do not confer susceptibility to other bacteria; however, the extent to which Neisseria has evolved complement inhibitory factors indicates the importance of this system for this group of pathogens [16]. Deficiencies in classical pathway proteins, such as C1q, increase susceptibility to encapsulated bacterial infections such as *Streptococcus pneumoniae* and *Haemophilus influenzae* since opsonization is required for efficient killing of these bacteria [17]. Similarly, alternative pathway defects in Properdin, Factor D, and Factor I heighten the risk of both meningococcal and encapsulated bacterial infections, since the amplification loop that the alternative pathway provides is important for both opsonization and MAC formation [18–20]. Thus, complement proteins play a crucial role in immune defence against bacterial pathogens [21–23].

Through the induction of humoral immunity, vaccination may mitigate the risk of infection in congenital or acquired complement deficiency. Patients with terminal complement pathway deficiencies can generate robust initial antibody responses to meningococcal vaccines, but protection may wane over time [24]. In a study of 53 individuals with complement deficiencies, antibody levels after vaccination with an unconjugated tetravalent polysaccharide vaccine were comparable to healthy controls when titres were assessed up to 6 months postvaccination. However, despite comparable antibody titres when measured at 6 months postvaccination, breakthrough infections with vaccine-strain serogroup Y occurred in two C8β-deficient individuals at 3.5- and 5-years following vaccination [25]. One explanation may be waning of the antibody response, since antibody titres at 6 months were reduced compared with 2 months postvaccination, however in the case of classical complement component deficiency and asplenia the risk of pneumococcal disease persists despite the presence of otherwise protective levels of antibody [26].

Therefore, an alternative explanation may be that serological correlates of protection need to be contextualized with downstream effector mechanisms in mind. In the absence of these downstream effector mechanisms, titres considered protective by ELISA in individuals without complement deficiency may not apply, and a functional assay such as a serum bactericidal or opsonophagocytic assay with patient serum may be more relevant. A vaccine in a patient with such an IEI may therefore be immunogenic, but not necessarily efficacious. This justifies the use of prophylactic antibiotics in patients with complement deficiencies, in addition to enhanced vaccine-response monitoring [21, 22, 27].Conjugate vaccines elicit stronger and more durable immune responses by inducing the complement-fixing IgG1 subclass, compared with predominantly IgG2 responses from polysaccharide-only vaccines, which fix complement less well [24]. Conjugate vaccines are now preferred for both complement-deficient as well as complement-replete individuals and highlight the importance of complement in controlling infections, particularly with encapsulated bacteria.

There are a growing number of therapeutics which interfere with complement cascade proteins. Therapeutic anti-C5 monoclonal antibodies (e.g. Eculizumab) are used in a variety of conditions such as atypical haemolytic uraemic syndrome and paroxysmal nocturnal haemoglobinuria and by design induce an acquired terminal pathway complement deficiency. This therapy has an associated increased risk of meningococcal infection, mandating meningococcal immunization prior to treatment [11]. Like individuals with genetic complement deficiency, incomplete vaccine-induced protection against Neisserial infection has been observed, with fatal vaccine-strain invasive meningococcal and gonococcal disease reported in eculizumab-treated patients despite having received conjugate immunization [28–33]. Together, patients with complement component defects are at increased risk of infection and enhanced vaccination strategies may be required to mitigate this risk, as well as antibiotic prophylaxis.

## Defects in intracellular immune signalling pathways and cytokine signalling

Signalling pathways, such as those initiated by Toll-like receptors (TLRs), are essential for detecting pathogens and inducing innate immunity. Defects in these pathways influence susceptibility to a variety of infections, including invasive pneumococcal disease [34] and also SARS-CoV-2 infection [35], among many bacterial, viral, and fungal infections.

Autosomal recessive defects in IL-1 receptor-associated kinase (IRAK)-4, myeloid differentiation factor (MyD)-88, and TLR/IL-1 receptor adaptor protein (TIRAP) [36] impair downstream signalling of IL-1 and TLR pathways leading to heightened susceptibility to bacterial infections, particularly *S. pneumoniae*, *Staphylococcus aureus*, and *Pseudomonas aeruginosa* [37]. Nuclear Factor kappa B (NF-κB), activated downstream of these proteins, is a critical transcription factor that upregulates cytokine production and other immune mediators. Defects in NF-κB and its regulator IκBα (**N**uclear **F**actor of **k**appa light polypeptide gene enhancer in **B**-cells **i**nhibitor **a**lpha) result in an even more profound immunodeficiency placing patients at risk of bacterial, fungal, and viral infections.

IRAK-4 and TIRAP deficiency are associated with impaired pneumococcal polysaccharide vaccine responses which could be explained by the observed loss of IgM^+^IgD^+^CD27^+^ B cells, reducing bacterial T-independent IgM responses to polysaccharide antigens [38, 39]. TLR-driven inflammation appears particularly crucial in early life, when adaptive immune responses are not yet fully developed; the risk of infection decreases with age as repeated infections drive compensatory humoral immunity [37] indicating that in certain circumstances, adaptive immunity may compensate for a defect in innate immune activation, supporting the use of vaccination in these individuals. Polymorphisms in TLRs are associated with differential responses to measles, mumps, rubella (MMR) [40] and influenza [41] immunizations, indicating their role in vaccine immunogenicity by driving initial innate immune responses. Outer membrane vesicle (OMV)-based vaccines rely on TLR signalling and impaired activation of innate immune signalling seen in mouse models of TLR4 deficiency demonstrates correspondingly impaired humoral immunity generated by the OMV meningococcal serogroup B vaccine Bexsero [42].

Loss-of-function mutations in type 1 interferon (IFN) receptors, or downstream signalling proteins, predispose individuals to severe viral infections, such as SARS-CoV-2 and influenza [43–46]. These defects also increase the risk of adverse events following live viral vaccines, such as yellow fever vaccination-associated viscerotropic disease or disseminated vaccine-strain measles, mumps, and rubella in patients with IFNα receptor, STAT1, and STAT2 deficiencies [47, 48]. Acquired autoantibodies targeting IFNα have also been linked to viscerotropic complications following yellow fever vaccination [49]. Notably, the prevalence of anti-cytokine autoantibodies increases with age, which may have implications for vaccination strategies that employ live vaccines in older individuals.

The role of interferon in immune responses to viruses and vaccines is, however, nuanced. In mouse models, transient impairment of type 1 IFN signalling appears to enhance live viral vaccine immunogenicity by increasing antigen load [50], and observations of enhanced IFN responses in children to viral infections with relatively reduced involvement of the adaptive immune response indicate a finely balanced system with interferon being required to prevent disseminated disease from live viral vaccines, but also some evidence to suggest that IFN-induced antiviral activity may suppress the development of long-lasting adaptive immune responses.

The recognition of IFN signalling in generating immune responses to mRNA vaccines has also been recognized. One of the primary challenges in early mRNA vaccine research was the innate immune recognition of the mRNA itself, which led to substantial reactogenicity. This reactogenicity was driven by TLR7, melanoma differentiation-associated protein-5 (MDA-5), and mitochondrial antiviral signalling protein (MAVS), key components of pathways detecting foreign RNA and leading to upregulation of IFN, among other cytokines. This issue was overcome by modifying mRNA to include 1-methylypseudouridine (m1ψ) in place of uridine, reducing innate immune recognition while maintaining effective translation [51]. An important question in mRNA vaccine development has been addressing how these vaccines achieve the necessary innate immune activation to elicit an adaptive response. Studies in mice demonstrated that lipid nanoparticles (LNPs) play the critical role in establishing germinal centres. This process relies on signalling via IL-6 but does not require MyD88 or MAVS, suggesting that neither TLR pathways nor intracellular RNA sensing are essential [52]. The role of residual TLR activity or interferon (IFN) signalling in human mRNA vaccine responses was explored in patients with deficiencies in innate immune sensing pathways, including TLR7, IRF7, and type 1 IFN receptors. These patients exhibited unimpaired vaccine responses, producing humoral responses comparable to those of healthy controls [53]. Understanding the mechanisms of innate immune activation by LNPs will be critical for refining this innovative vaccine platform. These insights offer opportunities to optimize mRNA vaccines for broader use, including in populations with dysregulated innate immune signalling [54].

The use of monoclonal antibodies targeting specific cytokines in immune-mediated inflammatory disorders represents a novel form of acquired immunodeficiency. Tumour necrosis factor alpha (TNF-α) is critical for the control of mycobacterial infection [55], and consistent with this, fatal disseminated *Mycobacterium bovis* bacille Calmette-Guérin (BCG) infection in infants has been reported following transplacental transfer of monoclonal anti-TNF-α antibodies used to treat maternal inflammatory bowel disease [56]. Accordingly, live attenuated vaccination against either bacterial or viral pathogens is recommended to be deferred in infants exposed to anti-TNF-α therapy until 6 months of age. TNF-α inhibition has been shown to reduce memory responses to SARS CoV-2 vaccination, suggesting an important role for this cytokine in either vaccine immunogenicity or the preservation of immunological memory [57]. In contrast, blockade of interleukin 1 (IL-1) did not generate differences in SARS-CoV-2 vaccine responses in patients with autoinflammatory diseases, highlighting complexity and/or redundancy within acute inflammatory cascades [58].

## Phagocyte defects

Deficiencies in neutrophil number or function predispose individuals to severe bacterial and fungal infections. Chronic granulomatous disease, caused by genetic defects in the oxidative burst machinery, results in heightened susceptibility to *S. aureus*, *Aspergillus*, and Mycobacterial infections, among others [59, 60]. Although the role of neutrophils in adaptive immune responses to vaccination is not well understood, recent studies suggest that neutrophils play a role in initiating the inflammatory response required for effective antibody production. This includes the secretion of B cell-activating factor (BAFF), which supports the development of robust humoral responses [61, 62]. Despite the importance of neutrophils, the absence of susceptibility of CGD patients to *S. pneumoniae* and intact adaptive immunity indicates that appropriate vaccine response can be achieved in the absence of certain phagocyte functions [63].

A significant concern for patients with phagocyte disorders, such as CGD, is the safety of live vaccines such as BCG and the *Salmonella typhimurium* vaccine Ty21a. One study reported that two-thirds of CGD patients who received BCG vaccination developed BCG-related disease, including lymphadenitis and pneumonia [64]. The ability of macrophages to successfully phagocytose and destroy bacteria is essential for preventing complications from live bacterial vaccines. The risk of vaccine-derived BCG infections extends to individuals affected by loss-of-function defects in pathways affecting Th1 immunity and the interaction between these cells and macrophage lineages (e.g. IFN-γ and receptors, STAT1, NEMO, IL-12, and IL-12 receptor, JAK1, ISG15, IRF8, etc) [65–67].

Together, knowledge of the importance of phagocyte defects and signalling defects resulting in impaired phagocyte activation have contributed to vaccine policy implications such as live vaccines, either bacterial or viral, being contraindicated in patients receiving Janus family kinase (JAK) inhibitors for haematological or immune-mediated inflammatory disorders [68].

## Defects of adaptive immunity

The core principle of vaccinology lies in the generation of immunological memory, enabling the immune system to mount qualitatively and quantitatively superior responses upon re-exposure to a pathogen. The adaptive immune system is central to this process, generating antigen-specific B and T cells in response to vaccine antigens. Upon subsequent exposure to the same pathogen, memory B cells rapidly differentiate into antibody-producing plasma cells, and memory T cells initiate a swift and robust cellular immune response. High-affinity antibodies, developed through somatic hypermutation and affinity maturation in germinal centres, neutralize pathogens efficiently, preventing infection and disease progression.

The success of vaccines depends on their ability to induce both humoral and cellular immunity, with high-affinity antibodies playing a critical role in neutralizing extracellular pathogens, while cellular immunity is essential for targeting intracellular infections. By mimicking natural infection without causing disease, vaccines harness these mechanisms to provide long-lasting immunity.

IEI can disrupt lymphocyte numbers, lymphocyte activation, and high-affinity antibody production at various stages, resulting in highly variable vaccine immunogenicity and concerns about the safety of live vaccines, particularly in individuals with defective cellular immunity.

Compared with IEI causing defects in cellular immunity, our understanding of antibody deficiency and its impact on vaccination is relatively well developed due to larger cohorts to examine and standardized antibody binding assays to measure humoral responses. However, there is recognition that antibody binding may not correlate as well with antibody function as it might in a healthy individual. Tests of antibody functionality such as complement fixation or neutralization are not in routine clinical use (Table 2). Similarly, tools for assessing T cell function remain largely confined to the research setting and so our understanding of the impact of T cell deficiency (either IEI or acquired) on vaccination is comparatively limited. Despite these limitations, important lessons have been learned, particularly regarding the safety of vaccination in these vulnerable populations.

## Antibody-deficiency

IEI predominantly affecting antibody production are the most common causes of primary immunodeficiency. These disorders can result from monogenic defects in B cell development, such as X-linked agammaglobulinemia (XLA) [69, 70], or other autosomal recessive forms arising from mutations in genes involved in B cell development such as Igα/β, µ heavy chain, and λ5 deficiencies. The most prevalent polygenic antibody-deficiency IEI is common variable immunodeficiency (CVID), defined by IgG levels two standard deviations below the age-appropriate reference range, combined with either low IgA or IgM, poor antibody responses to vaccination, age greater than 4 years, and exclusion of secondary causes of hypogammaglobulinemia [71–73].

Test vaccination is an essential component of diagnosing antibody deficiencies and determining eligibility for treatment with immunoglobulin replacement. The pneumococcal polysaccharide vaccine Pneumovax-23 (PPV-23) is the most frequently recommended test vaccine [74, 75]. Measuring pneumococcal-specific antibody levels before and after vaccination allows assessment of the antibody response magnitude. By definition, therefore, patients with antibody deficiency show inadequate responses to at least some vaccines. However, methodological variability in how anti-pneumococcal antibody titres are measured, along with heterogeneity among healthy individuals in their response to PPV-23, significantly complicates the interpretation and utility of test vaccination with unconjugated pneumococcal vaccines [74, 76]. In contrast, protein-containing or conjugated vaccines (e.g. tetanus toxoid) tend to elicit more consistent responses, reflecting differences in immunogenicity between vaccine platforms. Assessing responses to different vaccine types provides a nuanced understanding of immune compromise, helping to identify patients at the highest risk of non-response and infection.

The SARS-CoV-2 pandemic offered a unique opportunity to explore immunogenicity of mRNA and adenoviral vector vaccination in populations of individuals with IEI with no pre-existing antigen-specific immunity, and 54.8% of individuals with antibody deficiency in the COVID-19 in patients with antibody deficiency (COV-AD) study achieved seropositivity after two vaccine doses, compared with 100% of healthy controls. mRNA vaccines exhibited greater humoral immune responses than viral vector vaccines in this cohort, yet fewer than 10% of individuals produced neutralizing antibodies at levels comparable to controls [77]. After a third dose of vaccination, 76% of the COV-AD cohort had detectable anti-spike antibodies [78]. Within the group of antibody-deficient patients, there was significant variation in vaccine responses. For example, individuals with XLA, who have no detectable B cells, were unable to mount any serological response but developed SARS-CoV-2-specific T cells producing robust IFN-γ and cytotoxic responses, even surpassing that of healthy controls in some instances [79] with similar findings reported for influenza, hepatitis B, and tetanus vaccines [80–82]. However, while cellular immunity provides partial protection against severe COVID-19, the absence of humoral immunity in XLA patients contributes to prolonged SARS-CoV-2 viral carriage following infection [80]. Analogously, live oral poliovirus vaccination can lead to the establishment of chronic, replicative vaccine-derived poliovirus infection amongst those with humoral immune deficiency, along with vaccine strain rubella found in granulomatous lesions of antibody-deficiency individuals [83, 84]. Nevertheless, SARS-CoV-2 vaccination is partially efficacious at a population level amongst individuals with antibody deficiency [85].

These observations provided important pieces of evidence. Firstly, a significant proportion of antibody deficient individuals respond to mRNA or adenoviral-vectored vaccination.when by definition, these respond poorly to pneumococcal vaccination, typically with PPV23, suggesting novel vaccine platforms may offer the opportunity to improve vaccine efficacy among immunologically vulnerable where clinical need is greatest. Heterologous vaccination schedules, where viral vector and mRNA vaccine doses were sequentially combined, may further augment antibody and T-cell responses, as demonstrated in the OCTAVE-DUO study, a multi-disease randomized clinical trial to assess the efficacy of SARS-CoV-2 vaccination booster dosing strategies in patients with primary and secondary immunodeficiencies [86]. The number of doses required to generate and maintain immunological responses amongst immunodeficient patients is different to healthy controls [78, 87–92]; this should be considered when licensing studies are undertaken. Furthermore, even amongst those where vaccine-induced antibodies are detected, the functionality or quality of these antibodies may not be equivalent to those of healthy controls, or entirely reflective of the overall efficacy of vaccination. There remains a need to identify biomarkers that further stratify clinical risk of severe infectious disease within vulnerable populations so alternative infection prevention or treatment strategies can be deployed.

Whilst individuals with IEI affecting antibody production are rare, patients with haematological malignancy and autoimmune conditions are frequently treated with drugs that interfere with humoral immunity by targeting CD20, CD19, CD38, BTK, and BCMA, among others. Like XLA, drugs that deplete B cell populations prevent humoral immune responses to *de novo* antigens, although cellular responses may be maintained as demonstrated in the analysis of Rituxumab-treated patients vaccinated against SARS-CoV-2 [93, 94]. After drug washout, B cell recovery normally occurs, providing populations of emergent naïve B cells that can respond to vaccination. However, the timing of B cell recovery is variable depending on the underlying condition and co-administered drugs. As a result, long-lasting B cell aplasia and antibody deficiency can occur [93, 95, 96]. An exemplar of this is the profound B cell depletion found following treatment with CD19-targeted CAR-T cell therapy; impaired responses to vaccination occur, whilst B cell numbers are low, but there is restoration of vaccine responsiveness upon B cell reconstitution [97]. This highlights the importance of timing of vaccination in treatment schedules, emphasized by a study which found that patients treated with methotrexate generate higher titres of SARS-CoV-2-specific IgG if vaccinated during a temporary cessation of treatment [98].

Evidence from both IEI and phenocopies from immunosuppressive treatments indicates that the optimal type timing and dosing of vaccine may be different to immunocompetent individuals and should be tailored to achieve the best responses [99]. Furthermore, the absence of humoral immunity should not preclude the use of vaccinations as there is clear evidence that cellular immunity can remain intact.

## Combined immunodeficiency

Given the critical role of T cells in driving high-affinity antibody production, T cell immunodeficiencies invariably result in at least some degree of impaired antibody production. The most severe form, severe combined immunodeficiency (SCID), arises from defects in lymphocyte development, leading to profound T cell deficiency and, variably, B and NK cell deficiency depending on the underlying IEI. Patients with SCID are highly vulnerable to infections, and these conditions are near-universally fatal within 18 months without allogeneic hematopoietic stem cell transplantation or gene therapy [100, 101]. Despite their lack of an effective immunological response to vaccination, important insights into vaccination strategies can be derived from SCID patients.

The absence or paucity of T cells incurs a significant risk for patients with SCID from live vaccines given in infancy [102]. Vaccination with BCG is a notable example. Approximately 51% of SCID patients receiving BCG experience significant complications including developing disseminated infection [103]. Beyond SCID, BCG-osis has also been reported in a variety of other primary immunodeficiency diseases, typically with monogenic defects in the IL-12/IFN-γ signalling apparatus and those with CGD [104, 105]. The risk of death from disseminated BCG infection was significantly greater in infants receiving vaccination at less than 1 month of age compared with those vaccinated later. This, combined with implementation of newborn screening for SCID [106], has prompted the United Kingdom Joint Committee on Vaccination and Immunization to recommend delaying administering BCG immunization at 28 days to at-risk children by which time a result from SCID screening would be available. Internationally, BCG is frequently administered at birth in countries with high TB prevalence [106]. There is not international consensus with regards to the timing of BCG vaccination in countries with newborn SCID screening. The Australasian Society of Clinical Immunology and Allergy position statement advocates for continued antenatal BCG administration in at-risk populations [107]. Whilst debated [108], it is likely that the reduction in risk to SCID patients in countries with newborn screening from a delay in vaccination will outweigh the risk of additional cases of tuberculosis [109].

Di George (22q11.2 deletion) syndrome is associated with varying degrees of thymic aplasia which can result in a severe T cell lymphopenia, and in some cases, necessitate thymic transplantation [110]. Data on vaccine immunogenicity in this condition are limited, but several studies have retrospectively evaluated the safety of live attenuated viral vaccines. Based on these findings, clinical practice guidelines for patients with thymic development disorders have been established [111, 112]. These guidelines recommend live vaccine administration only for patients meeting specific criteria, including a CD4+ count ≥ 400 cells/mm^3^, CD8+ T cell count ≥ 200 cells/mm^3^, the development of a protective tetanus IgG response after dose 3, along with the additional guidance of assessing the presence of CD45RA^+^ T cells as a higher proportion of CD3+ CD4+ T cells than CD45RO^+^ [111]. In 22q11.2 deletion syndrome, the absolute number of T cells appears to be the most relevant metric for assessing vaccine safety. However, for other T cell defects that impact T cell function, additional considerations beyond simple T cell counts may be necessary, such as assessment of naïve T cell populations and T cell proliferation.

In addition to genetic causes of T cell deficiencies, various immunosuppressive drugs can impair T cell function and numbers, potentially affecting vaccine efficacy and safety. Medications such as calcineurin inhibitors (e.g. tacrolimus, cyclosporine) and antimetabolites (e.g. mycophenolate mofetil) have been associated with diminished T-cell responses to vaccination in some studies [113–115]. Similarly, corticosteroids, depending on dosage, route of administration, and duration, can impair T cell-mediated immunity and alter the balance of T cell subsets, reducing overall vaccine efficacy. However, as many underlying health conditions are independently associated with poor vaccine responses [116], the overall effect of immunosuppressive treatment on desired immune responses is nuanced.

Patients receiving high-dose immunosuppression and those with combined immunodeficiencies are at high risk of severe disease from vaccine-preventable infections and heightened risk for complications from live attenuated vaccines, such as MMR, and varicella vaccines due to suppressed T cell function [117]. Consequently, live vaccines are generally contraindicated in most individuals with IEI, many with secondary immunodeficiency and those with receiving high dose immunosuppressive treatment [68]. While inactivated vaccines are safer alternatives, their immunogenicity may still be suboptimal. Ideally, vaccine schedules should be tailored to the degree of T cell immunosuppression and immune recovery; however, this approach is limited by the lack of standardized and accessible diagnostics to comprehensively assess T cell function. While T cell enumeration is straightforward, more advanced assays such as phenotyping or functional ELISpot tests remain largely confined to research settings.

Strategies to optimize vaccine responses in these patients may include administering vaccines before initiating immunosuppressive therapy when feasible, using adjuvanted or additional vaccine doses and monitoring immune responses post-vaccination. The potential benefits of vaccination of patients with IEI must be balanced against potential risks from this approach including possible adverse events such as administration of live attenuated vaccinations to at-risk individuals.

## Implications for immunization strategies in immunocompromized

Immunization strategies for immunocompromized patients must address their heightened vulnerability to infectious diseases while navigating reduced vaccine immunogenicity and lack of immune correlates of protection for patients with IEI. Lessons from the SARS-CoV-2 pandemic underscore the potential of vaccines to provide partial protection, even in patients with severe immune deficiencies. Bespoke approaches, such as additional doses or heterologous schedules, have shown promise in enhancing immunogenicity in vulnerable populations.

For patients on immunosuppressive therapies, understanding vaccine immunogenicity is essential. Strategies such as timing vaccinations prior to initiating immunosuppression or administering booster doses to counter rapid antibody decline can enhance vaccine efficacy. Tailored schedules, such as delaying BCG administration in SCID screening, demonstrate how individualized planning improves safety and outcomes.

In cases where immunization is unlikely to provide sufficient protection, passive immunization serves as a critical alternative if appropriate patient groups can be identified. Immunoglobulin replacement therapy is a cornerstone for antibody-deficient patients, offering protection against many pathogens. Monoclonal antibody therapies and antimicrobial prophylaxis can bridge these gaps, providing targeted protection in high-risk individuals.

As history has repeatedly shown, vaccination has enormous potential to prevent death and disease. However, patients with impaired immune systems are often at greatest risk from infectious diseases yet derive the least benefit from standard vaccination strategies. Immunization in patients with IEI highlights the complexity of compensating for immune impairments, offering insights for broader cohorts, including those with secondary immunodeficiencies caused by underlying diseases or immunosuppressive therapies. These lessons guide efforts to minimize disease from ubiquitous pathogens, ensuring that the benefits of immunization extend to all populations.

## Data Availability

The source material in this review was obtained from publicly available peer-reviewed literature, accessible through databases such as PubMed, Web of Science, and Scopus. No original data were generated for this review; therefore, all data supporting the findings are readily available within the cited publications.

## Abbreviations

BCG: bacille Calmette-Guérin
COV-AD: COVID-19 patients with antibody deficiency
CVID: common variable immunodeficiency
IEI: inborn errors of immunity
IRAK: IL-1 receptor-associated kinase
LNP: lipid nanoparticle
MAVS: mitochondrial antiviral signalling protein
MD: myeloid differentiation
MDA-5: melanoma differentiation-associated protein-5
MMR: measles, mumps, rubella
MyD: myeloid differentiation factor
NF-κB: Nuclear Factor kappa B
OMV: outer membrane vesicle
SCID: severe combined immunodeficiency
TIRAP: TLR/IL-1 receptor adaptor protein
TLR: Toll-like receptor
TNF-α: tumour necrosis factor alpha
XLA: X-linked agammaglobulinemia
